# Macrophages and the microbiome in chronic obstructive pulmonary disease

**DOI:** 10.1183/16000617.0053-2024

**Published:** 2024-12-04

**Authors:** Karanjot K. Sandhu, Aaron Scott, Amanda L. Tatler, Kylie B.R. Belchamber, Michael J. Cox

**Affiliations:** 1 Department of Microbes, Infection and Microbiomes, Institute of Microbiology and Infection, School of Infection, Inflammation and Immunity, College of Medicine and Health, University of Birmingham, Birmingham, UK; 2 Department of Inflammation and Ageing, School of Infection, Inflammation and Immunity, College of Medicine and Health, University of Birmingham, Birmingham, UK; 3 Centre for Respiratory Research, School of Medicine, University of Nottingham, Nottingham, UK; 4 Biodiscovery Institute, University of Nottingham, Nottingham, UK; 5 NIHR Nottingham Biomedical Research Centre, Nottingham, UK; 6 These authors contributed equally

## Abstract

COPD is a heterogeneous disease of the lungs characterised by restricted airflow. Chronic inflammation and recurrent bacterial infections are known to be important driving factors in exacerbations of this disease. Despite a marked increase in the number of alveolar macrophages present in the lungs of COPD patients, there is evidence of reduced clearance of pathogenic bacteria, leading to recurrent infection, exacerbation and subsequent lung function decline. This is thought to be attributed to a defect in the phagocytic capability of both alveolar and monocyte-derived macrophages in COPD. In addition to this defect, there is apparent selectivity in bacterial uptake by COPD macrophages because certain pathogenic genera, such as *Haemophilus*, *Moraxella* and *Streptococcus*, are taken up more readily than others. The respiratory microbiome plays a key role in regulating the host immune response both in health and during chronic inflammation. In patients with COPD, there are distinct changes in the composition of the respiratory microbiome, particularly the lower respiratory tract, where dominance of clinically relevant pathogenic species is commonly observed. Whether there are links between these changes in the microbiome and dysfunctional macrophage phagocytosis has not yet been widely studied. This review aims to discuss what is currently known about these phenomena and to explore interactions between macrophages and the respiratory microbiome.

## Shareable abstract

**This review explores the interface between defective macrophage phagocytosis and distinct changes in the respiratory microbiome observed during COPD, whereby selective uptake of pathogens may be driving community structure.** https://bit.ly/3Mpj7kM

## Introduction

Chronic obstructive pulmonary disease (COPD) is a progressive, chronic inflammatory disease of the lower respiratory tract (LRT) predominantly initiated by the inhalation of noxious particles such as cigarette smoke. COPD is characterised by three underlying pathophysiologies, chronic bronchitis, small airways disease and emphysema, all of which lead to progressive airflow obstruction. Exacerbations of COPD, defined as the gradual worsening of symptoms, are linked to pathogenic infection of the LRT [1], as well as dysfunction of alveolar macrophages (AMs) [1, 2]. The lungs harbour a diverse community of microbial residents, termed the respiratory microbiome, which is implicated in COPD pathophysiology [3–5]. The microbiome comprises fungi, viruses and bacteria, although the focus of this review will be on the latter. This review considers potential links between AMs and the LRT microbiome, as well as wider host–microbiome interactions during COPD.

The use of terms to describe microorganisms and their pathogenesis is inconsistent between the microbiome and immunology fields. Here we use “pathogen” to describe organisms actively involved in a pathogenic process. “Pathobiont” will be used to describe those that may become involved in pathogenic processes, and here is equivalent to potentially pathogenic microorganisms, or carriage. Some common respiratory pathogens, *e.g.* non-typeable *Haemophilus influenzae* (NTHi) and *Streptococcus pneumoniae*, can be carried without harm and may be best described as pathobionts [6]. “Microbiome” is used here to describe all organisms present in the body site, along with their interactions with the host, and “microbiota” describes just the community of microorganisms. We are considering pathogens and pathobionts as part of the microbiota, not separate from it.

**FIGURE 1:**
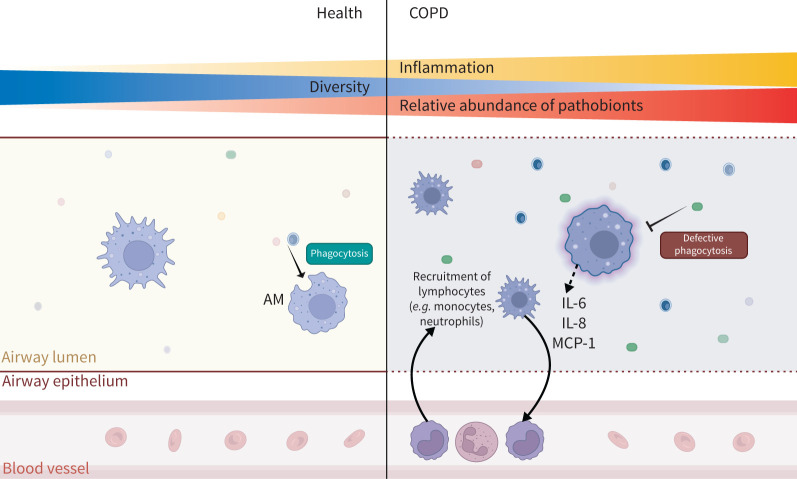
Defective phagocytosis and change in lower respiratory tract (LRT) microbiome dynamics in COPD. During chronic inflammation, alveolar macrophages (AMs) secrete pro-inflammatory cytokines, which recruit monocytes from peripheral blood vessels to aid clearance. In health, the airways are colonised with microorganisms (microbiota), including the presence of pathobionts. In COPD, increased inflammation causes the relative abundance of pathobionts to increase, while there is an overall reduced diversity of the LRT microbiota. Structural damage and environmental changes to the airway epithelium and lumen may also induce genotypic and phenotypic alterations in some pathobionts (green and blue), allowing them to become pathogenic and cause active infection. IL: interleukin; MCP: monocyte chemoattractant protein.

The interface between macrophages and the respiratory microbiome is important in maintaining airway health. In health *versus* COPD, there are distinct differences in both the composition of the respiratory microbiota and the phenotypes and functions of macrophages (figure 1), and their contributions to a pro-inflammatory environment are not mutually exclusive.

## Alveolar macrophages

The innate immune response plays an integral role in the clearance of pathobionts and in preventing infection in the lungs [7]. This complex system involves several host defence cells, including macrophages. AMs are a distinct, self-perpetuating subset of macrophages, functionally and phenotypically distinct from other tissue-resident macrophage populations [8]. AMs are constitutively resident in the alveoli, where they are directly exposed to the external environment, and to more microorganisms than other tissue-resident macrophages [9]. AMs are mediators of host defence, playing a key role in the initiation and resolution of the inflammatory cascade within the airways [7].

### Function in healthy lungs

As professional phagocytes, AMs act as a barrier against microbial pathogens in the airways. This is achieved through the release of signalling molecules including chemokines and cytokines, which modulate the local and systemic immune response [10]. While AMs can identify and eliminate microbial pathogens, it is not clear whether they do so selectively, ignoring the microbiota, or whether this is indiscriminate in healthy lungs. As described by the damage-response framework, microbial pathogenesis can be characterised as the outcome of host–microbe interactions, whereby changes in both the host and microbe can result in virulence and disease [11]. Bacterial species can migrate into the lower airways *via* micro-aspiration from the upper respiratory tract (URT) or through direct inhalation [12]. These changes in the ecological niche may lead to altered gene expression of otherwise commensal organisms to a more pathogenic phenotype, which may in turn initiate an inflammatory response [13].

AMs line the epithelia of the alveoli where they are ideally placed to sample inhaled air [14]. These cells populate the lungs during gestation, resulting in a long-lived population that self-renews in homeostasis during health. Throughout the lifetime, infection and inflammatory events may exceed the ability of this population to self-renew. This population may then be supplemented by the infiltration of monocytes from the periphery, which take on the AM-like phenotype [15]. This repopulation of the AM niche is especially important in COPD, in which chronic inflammation and infection may deplete the resident pool and an influx of monocytes is required to aid clearance of pathogens from the airways [16]. Macrophages from the periphery will not have had the same complex prior exposures as AMs and are likely to be pro-inflammatory [17]; we might expect them to respond to the microbiota differently.

Phagocytosis is initiated by the interaction between pattern recognition receptors (PRRs), expressed on the macrophage cell surface, with pathogen-associated molecular patterns (PAMPs), found on the surface of pathogens. Toll-like receptors (TLRs) are one example of PRRs that play a role in recognising bacteria and signalling secondary responses within the macrophage, such as actin remodelling. This allows pathogens to be taken up by the phagocyte and induces the release of pro-inflammatory cytokines and chemokines, which recruit other immune cells to the airways such as neutrophils and eosinophils. These cytokines include interleukin (IL)-1β, tumour necrosis factor (TNF)-α, monocyte chemoattractant protein 1 and IL-6 for monocyte recruitment, as well as IL-8 and leukotriene B4 (LTB4) for neutrophil recruitment [18]. Recruited monocytes differentiate into monocyte-derived macrophages (MDMs) to increase the macrophage pool, whereas neutrophils increase the phagocytic capacity of the airways [19, 20]. One of the most widely studied PAMPs known to activate macrophages is lipopolysaccharide (LPS), a cell wall component found primarily in gram-negative bacteria [21] that binds to TLR-4 on the macrophage cell surface to induce the activation of interferon regulatory factor 5, NF-κB and activator protein 1. Following phagocytosis, internalised bacteria undergo several mechanisms intended to kill and destroy the pathogen, including acidification and enzymatic degradation within the phagosome [22]. There are gram-negative pathobionts, such as *Neisseria meningitidis*, which are common members of the microbiota, and therefore have cell walls that also contain LPS [23]. These are apparently well tolerated by the host and avert phagocytic uptake, although the mechanisms for this outside the context of infection are still unclear; this regulation has been broadly attributed to crosstalk between microbial metabolites and host immune cells [24].

### Function during COPD

**FIGURE 2:**
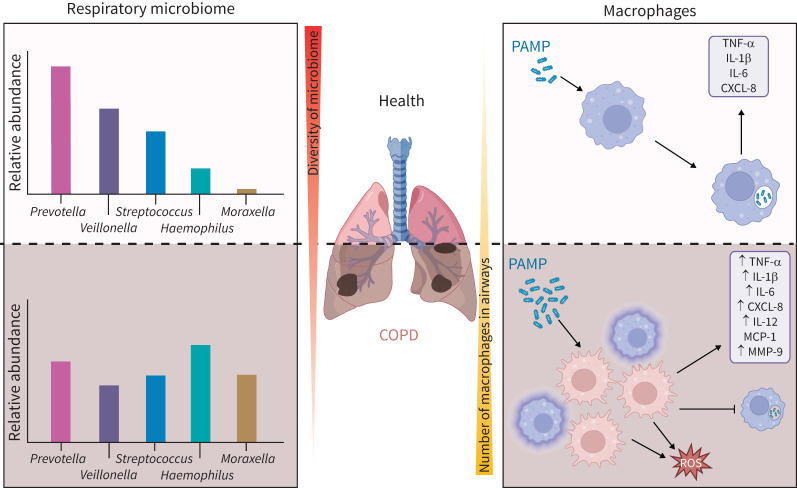
Differences in microbiome composition and macrophage function between health and COPD. There are distinct changes in composition of the lower respiratory tract microbiome in COPD compared to health, notably an increased relative abundance of *Haemophilus* and *Moraxella* genera. There are more macrophages in the airways during COPD and these exhibit a more pro-inflammatory phenotype and higher release of reactive oxygen species (ROS). COPD macrophages also have decreased ability to uptake pathobionts. PAMP: pathogen-associated molecular patterns; TNF-ɑ: tumour necrosis factor-α; IL: interleukin; CXCL: C-X-C motif chemokine ligand; MCP: monocyte chemoattractant protein; MMP: matrix metalloproteinase.

AMs are described as the orchestrators of COPD owing to the pivotal role they play in disease pathophysiology [25]. Despite an increase in the number of macrophages in the COPD lung, continuous colonisation with pathogens and pathobionts is observed, resulting in a chronic pro-inflammatory environment [26]. This may be due to impaired phagocytosis of microorganisms as well as expression and release of pro-inflammatory cytokines, observed in both AMs and MDMs from COPD patients, resulting in an overall inability to clear infection (as summarised in figure 2). Defective phagocytosis has been shown with both NTHi and *S. pneumoniae* [27], which are common exacerbation-causing bacteria, but also with other pathogens including fungi, such as *Aspergillus fumigatus*, indicating a shared defective mechanism [28]. It should be noted that this phagocytic defect is specific to microorganisms because exposure of COPD macrophages to inert polystyrene beads evokes a normal phagocytic response [29]. In phagocytosis experiments, it is important to consider what has been compared; pathogens are not frequently compared to pathobionts or the microbiota and therefore studies do not always resolve the differences between tolerated and non-tolerated microorganisms [30].

Defective phagocytosis is further associated with exacerbation frequency in COPD [31], strengthening the hypothesis that macrophages drive inflammation in the lungs. Recent studies have aimed to identify the underlying mechanisms behind this defective phagocytosis. Belchamber *et al.* [32] found that exogenous oxidative stress, such as hydrogen peroxide, reduced phagocytosis of heat-killed NTHi and *S. pneumoniae* by AMs in both COPD patients and healthy nonsmokers, indicating a mechanism that may explain the relationship between smoking and COPD. This same study showed that COPD MDMs displayed impaired mitochondrial function after phagocytosis, implicating mitochondrial defects as a mechanism of macrophage dysfunction. Bacteria such as *Legionella pneumophila* have also been shown to modify host mitochondrial function to their advantage, linking these concepts [33].

Macrophage numbers in the lungs of COPD patients are elevated compared to in healthy nonsmokers, as indicated in lung tissue from patients with Global Initiative for Chronic Obstructive Lung Disease (GOLD) 3 and 4 and can reach up to a 25-fold increase [26]. Increased macrophage numbers correlate with the severity of airflow limitation [34, 35], suggesting a direct link between macrophages and disease pathogenesis. There is an apparent paradox between increased macrophage abundance and a greater bacterial load during chronic inflammation (figure 1). This may be attributed to the defective uptake ability observed in COPD macrophages, which in turn leads to higher risks of infection and disease progression [36].

Matrix metalloproteinases (MMPs) are a family of structurally related proteinases that degrade components of the extracellular matrix and are involved in tissue remodelling and maintenance. MMP-12, also known as macrophage elastase, is elevated in the sputum of COPD patients [37], with human lung tissue from COPD patients expressing more MMP-12 than tissue from healthy controls [38, 39]. The release of MMP-12, as well as MMP-2 and MMP-9, are stimulated by exposure to irritants such as cigarette smoke and play a significant role in the pathogenesis of cigarette smoke-induced emphysema in mice [40–42]. Although MMPs are thought to perform their functions extracellularly, intracellular pools of MMP-12 have been found in quiescent murine macrophages, suggesting that MMPs also contribute to bacterial clearance [43]. However, no studies have identified the intracellular role of MMPs within AMs. Their release has been found to influence how effectively bacteria are cleared by AMs, as per the study conducted by Houghton *et al.* [44], where MMP-12^−/−^ mice showed an impaired ability to clear *Staphylococcus aureus* and increased mortality from both gram-positive and gram-negative infections. Although the inhibition of MMP-12 may seem like a potential intervention to lessen the burden of chronic inflammation in COPD patients, it could lead to a considerably increased risk of bacterial infection.

Some pathogens can either avoid or exploit intracellular defence mechanisms once internalised into a macrophage and instead use host cells to survive and replicate within the phagosome. There are several examples of bacteria in the respiratory tract that can continue to thrive within immune cells post-phagocytosis, such as *Mycoplasma pneumoniae* and *L. pneumophila* [27, 45]. Intracellular survival strategies are used by some bacteria as a means of disseminating and causing systemic infection within their host, which can in turn infect other organs. NTHi strains have been shown to survive and persist within macrophages, within both murine and human *in vitro* models*,* associated with an increase in systemic disease [46, 47]. Similarly, *S. aureus* can survive within phagosome compartments once phagocytosed by macrophages through inhibition of autolysosome fusion, as well as exhibiting altered activity and exotoxin production [48]. In addition, Bewley *et al.* [49] showed that COPD AMs express elevated levels of myeloid cell leukaemia 1, an anti-apoptotic protein that modulates mitochondrial oxidative phosphorylation, which was linked to a reduced delay of intracellular bactericidal activity upon infection with *S. pneumoniae*.

There is variability in the methodological approaches used to study defective phagocytosis in COPD. While heat-killed bacteria have been used in many assays owing to ease of use [50, 51], live bacteria have different properties compared to nonviable bacterial cells. For example, the ability to switch gene expression and adapt to changes in the local environment can either cause or contribute to their evasion of phagocytosis [52]. To investigate the phagocytosis of live bacteria by lung macrophages, some studies have used fluorescently labelled bacteria using fluorophores such as fluorescein isothiocyanate (FITC) [29, 53]. These methods are more representative of the phagocytic process *in vivo* than the use of heat-killed bacteria. There are still some limitations to consider: FITC binds covalently to the N-terminus of outer membrane proteins of bacteria, which can influence their metabolism and virulence, as seen with the respiratory tract pathogen *Bordetella pertussis* [54]. In the context of the microbiome, there are added complexities with using labelling approaches because these covalent modifications may affect inter-microbial interactions.

**FIGURE 3:**
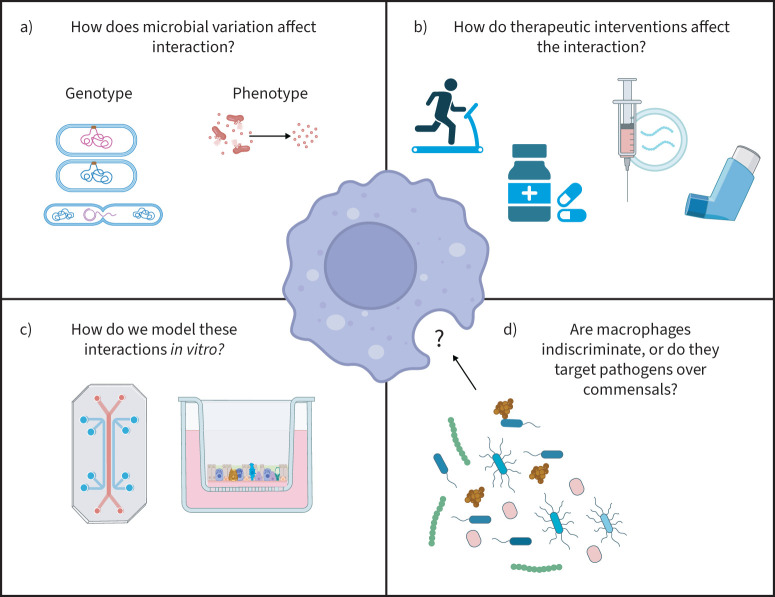
Current knowledge gaps of macrophage–microbiome interactions in the airways. Key areas that may affect this interface include a) microbial variation such as genetic differences, *i.e.* strain-level variation and transfer of genetic material between strains, and species and phenotypic differences, *e.g.* the release of different metabolites and/or toxins from the species present in a host; b) response of the microbiome and macrophage function to existing therapeutic interventions for COPD, *e.g.* inhaled and orally administered corticosteroids or antibiotics, pulmonary rehabilitation and vaccinations; c) the methods used to study the host–macrophage interface, which are important and should mimic the *in vivo* environment as closely as possible, *e.g.* microfluidic devices or “lung on chip” and air–liquid interface models, with longitudinal studies also important for understanding macrophage–microbiome relationship over time; and d) potential selectivity of macrophages during health and disease and whether certain species or strains are targeted more readily than others.

Other well-established approaches to studying host–microbe interactions in the airways include the use of air–liquid interface models including host immune cells [55]. These co-culture models allow for the culturing of host cells alongside live bacteria, which is a more physiologically relevant approach than single-cell culture models. Similarly in the context of the gastrointestinal human–microbe interface, Shah *et al.* [56] have developed the modular microfluidics-based human–microbial co-culture model (HuMiX), which overcomes some of the limitations posed by air–liquid interface. This methodology is promising and translational, whereby similar “lung on chip” microfluidic models could be used (figure 3c). This highlights the importance of developing methodologies that better mimic *in vivo* conditions while still retaining control over larger numbers of variables. Rapid, shallow sequencing and molecular methods may also allow for tracking of complex populations in such models [57].

## The respiratory microbiome

### In healthy lungs

The composition of the respiratory microbiota is believed to be dependent on three main factors: migration of microbes into the airways; elimination through host defence mechanisms such as mucociliary clearance, immune cells or the coughing reflex; and alterations in the microenvironment of the microbiome, including the structure of the airways [58]. This has been described as the adapted island model and, in the healthy lung, this dynamic equilibrium maintains a homeostatic balance of the microbiota in different locations along the respiratory tract, namely the oropharynx, the bronchial tree and the lung [59]. Parameters such as nutrient availability, oxygen tension, pH and presence and activation status of inflammatory cells such as macrophages are likely to determine the community composition of an ecological niche within the lungs. Mechanisms are yet to be fully described, and the relative importance of these and yet-to-be-discovered parameters is unknown.

While there are distinct differences in the microenvironments between the URT and LRT, *e.g.* colder surface and wall temperatures of the upper trachea and bronchial tree compared to the core body temperature, there is a clear overlap between the microbiota residing in the oral cavity and the lungs of healthy individuals [60]. Although the LRT exhibits a lower species richness compared to the URT, the microbiota found in both locations are closely linked [3, 60, 61]. In healthy individuals, the respiratory microbiota is populated predominantly with Bacteroidetes, Firmicutes and Proteobacteria phyla, with the most frequently identified genera being *Streptococcus*, *Prevotella* and *Veillonella* [3].

The respiratory microbiome has been shown to be involved with regulation of the host immune response. For example, specific pathogen-free mice demonstrated decreased reactive oxygen species (ROS) release from AMs, which in turn impaired antibacterial activity leading to reduced clearance of pathobionts in the airways and alveolar spaces [62, 63]. Moreover, there is an exclusive correlation between the variations in an individual's lung microbiota and the baseline concentration of the pro-inflammatory cytokine IL-1α, part of the TNF-α activation pathway that plays a role in modulating the immune response [63]. The LRT microbiota also plays a role in the activation and recruitment of anti-inflammatory macrophages, regulatory T-cells and tolerogenic dendritic cells [64].

Bacterial metabolites also form an important part of the LRT microbiome and have several effects on the host, particularly on cell signalling pathways. The release of ROS and nitric oxide synthase from bacteria has been shown to have damaging effects on host DNA as well inhibiting signalling pathways [65, 66]. Additionally, some common bacteria found within the airways, such as *Megasphaera*, *Streptococcus mutans* and *Streptococcus sanguis*, generate short-chain fatty acids that inhibit cytokine production and inflammation following stimulation of macrophages by LPS [67, 68].

The Anna Karenina principle also seems to apply to the respiratory microbiota [69]. Referencing the first line of Tolstoy's *Anna Karenina*, happy microbiomes are alike, whereas unhappy microbiomes are unhappy in their own way. There is greater variability between the respiratory microbiota composition in people with COPD than in people with healthy lungs.

### The microbiota in COPD

There is a microbiota in the airways in COPD [70] and it differs in composition from that of the healthy LRT [5]. The dominating phylum changes from Bacteroidetes towards Gammaproteobacteria, a class that includes pathobionts such as *Pseudomonas aeruginosa* and *H. influenzae*, which are increasingly abundant in the airways of COPD patients [71]. During stable COPD, the most prevalent genera were *Prevotella*, *Veillonella* and *Streptococcus*, as determined by 16S rRNA sequencing of COPD sputum samples [72]. According to a study by Erb-Downward *et al*. [73], the main taxa reported in the lungs of patients with COPD include *Pseudomonas*, *Veillonella*, *Prevotella*, *Fusobacterium*, *Haemophilus* and *Streptococcus* species. Of these, *P. aeruginosa, H. influenzae* and *S. pneumoniae* are well-described pathobionts, which are found to have a greater bacterial burden during acute COPD exacerbations [74].

A link between the severity of COPD and reduced diversity of the respiratory microbiome has also been established. Different community compositions can lead to inflammatory phenotypes. Sethi *et al.* [75] found that the acquisition of a pathobiont, which is not already present as part of the individuals’ normal microbiota, increases the risk of exacerbation in COPD patients. The isolation of a newly acquired strain of *H. influenzae*, *S. pneumoniae* or *Moraxella catarrhalis* in the sputum of 81 COPD patients was associated with a significant increase in the risk of exacerbation, suggesting that these genera have a causative role in COPD severity [76].

Diversity of the respiratory microbiome is also an important factor in immune regulation, particularly in the context of chronic inflammation. Jubinville *et al.* [77] demonstrated that patients with GOLD 3 COPD had a lower community richness and higher species evenness. This was determined through microbial characterisation of sputum samples from nine COPD patients at both stable and exacerbation states. Although this was a relatively small sample size, there have been similar findings in studies with larger cohorts. For example, Wang *et al.* [78] analysed the temporal variability of the lung microbiome using sputum samples from 281 COPD patients, at both baseline and exacerbation, from the COPDMAP study across three UK clinical centres. By performing a multivariate analysis, they identified a significant correlation between increased expression of C-reactive protein, an inflammatory marker for COPD prognosis, and both ɑ- and β-diversity at the phylum level across all samples. Dominance of a single pathogen in a sample lowers the diversity and so these drops in ɑ-diversity may represent the acquisition of a novel pathogen or the emergence of a pathogen from the microbiota [79]. This highlights that there are distinct alterations of the respiratory microbiome during COPD (figure 1).

## Respiratory microbiome and defective phagocytosis

This dynamic relationship between an increased abundance of pathogens in the respiratory tract and the progression of airway damage during COPD has also been described by a phenomenon widely known as the “vicious circle hypothesis” [76]. While this hypothesis primarily refers to pathogens, it is important to consider that changes in the environmental conditions within the lungs and respiratory tract will have a wider effect on the microbiota, and to consider how this might modify the vicious circle hypothesis. Factors that contribute to this sustained cycle of damage include the release of bacterial metabolites and virulence factors as well as increased expression of PAMPs, thus leading to a “vicious cycle” of pro-inflammatory response mechanisms by AMs and a decline in lung function [78, 80]. As discussed previously, the expression of PAMPs is not exclusive to pathogens or pathobionts and these are commonly possessed by non-pathogenic, commensal members of the microbiota. It is difficult to distinguish whether the increased inflammation and host defence mechanisms or altered microbiome are causes or consequences of each other, because both factors can be described as a driving force for one another.

Commensal bacteria are essential for homeostasis throughout the human body with regards to maintaining metabolic conditions. Macrophages can discriminate between “self” and “non-self” signalling molecules; however, this idea becomes more complex as we attempt to understand the lack of immune response to commensal bacteria, which identify as non-self. Because both pathogens and commensals can share the same signalling molecules and antigen, it raises the question as to whether the microbiome plays a role in this differentiation. It is important to note that pathogenicity is not always a trait possessed by a microbial organism, rather changes in the environmental conditions as well as host–microbe and microbe–microbe interactions can lead to pathogenicity. This has been studied extensively in the gut, where commensals such as *Helicobacter pylori*, which are part of the normal flora of an individual, can also cause gastritis [11]. The ability of bacterial organisms to undergo phase variation in response to the slightest changes in host physiological conditions makes it difficult to distinguish between what may be described as a pathogen or a member of the microbiome (figure 3a) [81].

The presence of *S. aureus*, commonly found to reside in the URT, has been found to have a protective effect against a lethal inflammatory response in the lungs following challenge with influenza virus, compared to specific pathogen-free mice [82]. According to this study, *S. aureus* mediates the recruitment of CCR2^+^CD11b^+^ blood monocytes into the alveoli, which then differentiate into macrophages that exhibit an anti-inflammatory phenotype. The release of anti-inflammatory cytokines and inhibitory ligands can prevent lethal inflammation caused by influenza infection. This priming effect demonstrated by *S. aureus* shows one way in which the respiratory microbiome can aid the host's immune response in preventing infection of the airways and the subsequent progression of disease.

Subtle variations in the genotype and phenotype of pathogenic bacterial strains between individuals may also be a contributory factor in defective phagocytosis by lung macrophages. In *S. pneumoniae,* some multi-locus sequence types (STs) have been associated with disease more than others, although this is not specific to COPD [83]. It has been found that strains of the same ST and capsule serotype exhibit important genetic and phenotypic differences [84]. Ackland *et al.* [85] found that MDM response was altered with different clinical strains of NTHi in healthy individuals, in whom the ST14 strain induced an increase in the expression of IL-10 and NF-κB compared to ST201. Similarly, it has been found that clinical isolates of NTHi from COPD patients can adapt their genome to enhance their persistence in the respiratory tract during chronic inflammation [86]. Some of the genetic adaptations observed had direct links to immune escape and antigenic variation, such as an increase in single sequence repeats of sialyltransferase, which aid molecular mimicry to evade the host immune response. It is possible that these differences in clinical isolates of some pathogenic strains may be a driver behind defective phagocytosis by COPD macrophages. However, there is a lack of research into the interface between these individual-level strain variations and, specifically, how these contribute to defective phagocytosis by macrophages during COPD.

### Therapeutic targeting of macrophages and the microbiome

Do macrophages control clearance, or does pathogen evasion drive this interaction? Are pathobionts and the microbiota involved and how do they influence this process? Can these interactions also be targeted therapeutically? Macrophages may be a useful therapeutic target in COPD. Manipulation of phagocytosis, phagocytic receptors and the release of signalling molecules or changing the phenotype of macrophages may improve their function.

A common treatment for COPD is inhaled corticosteroids (ICS) in combination with bronchodilators [87]. ICS reduce exacerbations and improve lung function by suppressing airway inflammation through the activation of anti-inflammatory genes as well as the inhibition of inflammatory cells, such as macrophages [88]. The use of ICS also influences changes in the microbiome through both the promotion and the inhibition of intracellular persistence of *P. aeruginosa* and *H. influenzae*, respectively [89, 90]. Leitao Filho *et al.* [91] have found that the use of combined salmeterol and fluticasone in patients with stable COPD decreased abundance of *Haemophilus.* Other studies have reported a decrease in the ratio of Proteobacteria:Firmicutes following the use of ICS [87], although Belchamber *et al.* [92] reported no changes in macrophage phagocytosis after budesonide and fluticasone treatment. Overall, the effect of ICS on both macrophage immunology as well as microbiome composition appears to vary depending on subject cohorts, sampling methods and study design (figure 3b) [93].

Macrophages have been targeted using the antibiotic azithromycin, which inhibits bacterial protein synthesis. While an *in vitro* study found that azithromycin did not alter phagocytosis of *H. influenzae* in MDMs [29], some *in vivo* studies have shown that administration of low-dose azithromycin significantly improves bacterial phagocytosis by both AMs and MDMs in COPD patients [94, 95]. Although these changes were not directly attributed to changes in receptor expression, previous studies have shown that azithromycin increases levels of the mannose receptor, which is involved in bacterial phagocytosis [94]. This suggests that the pro-phagocytic effects of azithromycin could be a potential therapy for COPD patients to improve clearance by macrophages. Further research is needed to investigate the biological basis of these effects, because the work made use of heat­-killed *Escherichia coli* and polystyrene beads rather than pathogens, pathobionts or other members of the LRT microbiota (figure 3c).

Furthermore, Vecchiarelli *et al.* [96] showed that the antioxidant *N*-acetylcysteine improves phagocytosis by monocytes of *Candida albicans* in COPD; however, its oral administration did not improve AM antifungal activity. Sulforaphane, an activator of nuclear erythroid-related factor 2, has also been shown to restore *H. influenzae* recognition and uptake by COPD macrophages [97], by inducing greater expression of macrophage receptor with a collagenous structure (MARCO) receptors [98]. Despite these promising targets, another study showed no improvement in AM or MDM phagocytosis after exposure to p38, mitogen-activated protein kinase 1, Pi3kinase or rhodopsin kinase inhibitors, suggesting that these pathways are not key in mediating this defect [99]. Targeting other aspects of macrophage function, including mitochondrial dysfunction, has the potential to restore AM phagocytic function and requires further study [32].

Antibiotics are often used to aid the clearance of airway infection during exacerbation episodes. The effects of antibiotic use on the composition of the microbiome should also be acknowledged. While azithromycin has been proven to be effective in the clearance of exacerbation-associated pathogens such as *S. pneumoniae*, *H. influenzae* and *M. catarrhalis*, it has broad-spectrum activity against many gram-positive and gram-negative species [100]. Therefore, its administration is likely to cause inevitable depletion of the respiratory microbiome, as observed in patients with asthma [101], where although there is a reduction in the abundance of pathogens associated with asthma, its use is also associated with an overall decrease in bacterial richness. The specific effects of macrolide therapy on microbial colonisation of the airways during COPD are yet to be explored (figure 3d); however, given that the pathogenic genera for these two diseases overlap, it is likely that a similar effect will be observed.

Vaccinations are another mode of preventing infections during COPD, whereby the pneumococcal vaccine has been widely used to stimulate a humoral response against the *S. pneumoniae* capsular polysaccharides [102]. Efforts are also being put towards the development of vaccination therapies against other respiratory pathogens such as NTHi, and preliminary trials have found an overall decrease in rates of exacerbation [103]. Other studies have investigated the NTHi outer protein D as a potential candidate for vaccination against NTHi strains [104]. In the context of host–microbiome interactions, the effects of both bacterial and viral vaccinations on the function and composition of the respiratory microbiome are important to consider and require further study.

Furthermore, there is a need for more longitudinal analyses of macrophage phagocytosis, given that Singh *et al.* [31] found that there were no changes in phagocytic ability of COPD macrophages over the duration of a year. Longitudinal studies would also help improve the understanding of patterns of phagocytosis during health and disease and account for variability of sampling across studies and experimental models.

Targeting the microbiome as a therapeutic approach to improve the survival and quality of life of COPD patients has also been investigated. A commensal species, *Rothia mucilaginosa*, that predominantly resides in the oral cavity has been detected in the LRT of patients with chronic respiratory diseases and is associated with inhibition of pathogen-induced inflammation [105]. Both *in vivo* and *in vitro* studies have shown that the presence of *R. mucilaginosa* in sputum samples from patients with bronchiectasis and COPD is linked to reduced levels of MMPs as well as inflammatory cytokines IL-8 and IL-1B. Similarly, some commensal microbes can inhibit pathogenic colonisation, such as the ability of *Staphylococcus epidermidis* to prevent biofilm formation of *S. aureus* through the secretion of a serine protease [106]. Budden *et al.* [107] have more recently discovered the benefits of faecal microbiome transplantation in alleviating hallmark symptoms of COPD, including a reduction in macrophages in bronchoalveolar lavage fluid of mice following cigarette smoke-induced inflammation.

Additionally, Yan *et al.* [108] reported that intranasal treatment of mice with *Lactobacillus salivaris* and *L. oris* increased indole-3-acetic acid levels in the airways, which in turn increased IL-22 production by alveolar and interstitial macrophages in mice. IL-22 is known to have protective effects in the lungs, including defence against bacterial and viral infections [109]. This indicates the potential of manipulating the microbial and metabolite composition of the airways to reduce infection-induced inflammation during COPD (figure 3a). It is important that these studies are validated in alternative models, because the majority of these studies have been performed in murine models, which are known to be considerably different to human lungs [110]. Overall, further understanding of macrophage–bacteria interactions in health and COPD may lead to the identification of novel therapeutic agents that could improve macrophage clearance of bacteria, and thus reduce exacerbations of this disease (figure 3b).

## Conclusion

Understanding of the interactions between macrophages and the microbiome is at an early stage, based largely on pairwise interactions rather than networks of bacterial and host cells, using models that do not attempt to replicate *in vivo* conditions. There are still gaps in the literature regarding potential links between the selectivity of AMs for certain microbes over others both prior to and during chronic inflammation and how changes in the respiratory microbiome may play a role in this (figure 3d). Given the roles of both macrophages and the microbiota in health and disease, there is excellent scope for the development of therapeutic and intervention strategies to improve bacterial clearance by lung macrophages and to help reduce the progression and exacerbations of COPD. Further research is required to determine whether these approaches should be targeted toward macrophage dysfunction, manipulation of the microbial composition or both to improve patient outcomes. To reference *Anna Karenina* again, ferreting in one's lungs, one often ferrets out something that might have lain there unnoticed.

**Questions for future research**

- Do COPD macrophages exhibit a preference for certain pathobionts over others?
- Do macrophages build tolerance to clinically relevant pathobionts during health, which may drive their selective uptake during disease?
- Can the community structure of the respiratory microbiome be manipulated to improve uptake of pathogens by COPD macrophages?
